# Mapping whole genome shotgun sequence and variant calling in mammalian species without their reference genomes

**DOI:** 10.12688/f1000research.2-244.v2

**Published:** 2014-02-10

**Authors:** Ted Kalbfleisch, Michael P Heaton

**Affiliations:** 1 Department of Biochemistry and Molecular Biology, School of Medicine, University of Louisville, Louisville, KY, 40202, USA; 2 Intrepid Bioinformatics, Louisville, KY, 40202, USA; 3 USDA Meat Animal Research Center, Clay Center, Nebraska, 68933, USA

## Abstract

Genomics research in mammals has produced reference genome sequences that are essential for identifying variation associated with disease.  High quality reference genome sequences are now available for humans, model species, and economically important agricultural animals.  Comparisons between these species have provided unique insights into mammalian gene function.  However, the number of species with reference genomes is small compared to those needed for studying molecular evolutionary relationships in the tree of life.  For example, among the even-toed ungulates there are approximately 300 species whose phylogenetic relationships have been calculated in the 10k trees project.  Only six of these have reference genomes:  cattle, swine, sheep, goat, water buffalo, and bison.  Although reference sequences will eventually be developed for additional hoof stock, the resources in terms of time, money, infrastructure and expertise required to develop a quality reference genome may be unattainable for most species for at least another decade.  In this work we mapped 35 Gb of next generation sequence data of a Katahdin sheep to its own species’ reference genome ( *Ovis aries* Oar3.1) and to that of a species that diverged 15 to 30 million years ago ( *Bos taurus* UMD3.1).  In total, 56% of reads covered 76% of UMD3.1 to an average depth of 6.8 reads per site, 83 million variants were identified, of which 78 million were homozygous and likely represent interspecies nucleotide differences. Excluding repeat regions and sex chromosomes, nearly 3.7 million heterozygous sites were identified in this animal vs. bovine UMD3.1, representing polymorphisms occurring in sheep.  Of these, 41% could be readily mapped to orthologous positions in ovine Oar3.1 with 80% corroborated as heterozygous.  These variant sites, identified via interspecies mapping could be used for comparative genomics, disease association studies, and ultimately to understand mammalian gene function.

## Introduction

As the price per base for next generation sequencing continues to fall, sequencing projects that are broad in scope become possible for research groups with modest budgets. As a result, research tools and approaches that once required large consortia ^1– 3^, may now be used by small groups of collaborators or even independent labs. Although high throughput technology has been democratized, formidable impediments remain that prohibit researchers whose work is not in human, model human, or agriculturally important species from realizing its benefits. Specifically, sequence data, once produced, is mapped to a reference genome for the species of the subject under investigation. The 10ktrees ^4^ project describes the phylogenetic relationship of 299 even-toed ungulates. Of these, only cattle, swine, sheep, goat, water buffalo, and bison have annotated reference genomes. For the other species a reference genome has not been built, and will likely not be built for another decade or more.

The goal of this study is to investigate whether or not an even-toed ungulate could benefit from the reference genomes of the few member species that do have them. To test this, one lane of paired-end Illumina sequence data (~35 billion bases) for a Katahdin ram was generated and mapped to its ovine reference assembly Oarv3.1 ^5^ and to the bovine reference assembly UMD3.1 ^6^. The variants measured for the Katahdin ram vs. UMD3.1 demonstrate the wealth of information that can be derived from an interspecies mapping. The majority of these variants are homozygous, and for the most part represent species-specific differences. Any heterozygous variant plausibly represents an intraspecies variation present in sheep. Approximately 78 million homozygous, and 3.6 million heterozygous variants were identified for this ram in non-repeat regions of the cattle genome (which excluded the X chromosome; chrX). Mapping the same dataset to Oar3.1 corroborated more than 1.2 million of the heterozygous variants (>80% of what could be checked, see Table 1). This result suggests that high throughput sequence data for any of the distantly related even-toed ungulates may be mapped to the reference genomes of related species that have annotated references for variant discovery, comparative genetics, and even, perhaps genotype-phenotype association studies.

**Table 1.** List of the number of variants identified in UMD3.1 for which a corresponding position could be identified in Oar3.1, and of these the number of variants whose genotype was corroborated (80.3%) vs. Oar3.1. No variants identified on the X-chromosome of either reference were included in these totals.

Total UMD3.1 Hets not in Repeat Regions (NR)	3,672,099
Total UMD3.1 NR Hets with Corresponding OAR3.1 position	1,524,297
Total UMD3.1 NR Hets with GT Corroborated in OAR3.1	1,224,642

Results are presented here that include the variants identified for the animal against the bovine genome, as well as those corroborated by mapping to the sheep genome. Additionally, the mapped data sets (binary alignment map files) for this sheep are made available for inspection by researchers interested in analyzing the findings of this study for loci most relevant to their work. The datasets are stored in the data management system developed and maintained by Intrepid Bioinformatics, and may be viewed in a version of the Integrative Genomics Viewer ^7^ modified to use their web service application programming interface ( https://sourceforge.net/projects/intrepidbioinfo/), the UCSC Genome Browser ( http://genome.ucsc.edu) ^8^, or dragged and dropped into a number of other analytical tools such as SAMtools ( http://samtools.sourceforge.net) ^9^. This data set, and its direct access is designed to benefit researchers interested in comparative genomics, disease association studies, and ultimately understanding mammalian gene function ^10^.

## Methods

### Ethics statement

Prior to their implementation, all animal procedures were reviewed and approved by the care and use committees at the United States Department of Agriculture (USDA), Agricultural Research Service (ARS) Meat Animal Research Center (USMARC) in Clay Center, Nebraska.

### Reference sheep sequenced

The DNA used for whole genome shotgun sequencing (WGS) was from a Katahdin ram that is part of a U.S. sheep reference panel (USMARC animal number 200008100). The USMARC Sheep Diversity Panel version 2.4 (MSDPv2.4) consists of 96 rams from nine breeds, a composite population, and one Navajo-Churro: Dorper, White Dorper, Dorset, Finnsheep, Katahdin, Rambouillet, Romanov, Suffolk, Texel, USMARCIII composite (1/2 Columbia, 1/4 Hampshire, and 1/4 Suffolk ^11^), and one Navajo-Churro ram as previously described ^12^. For sequencing, ram 200008100 was chosen simply for its breed type (Katahdin) and because it sired numerous progeny in the research flock.

### Katahdin ram genotypes from the ovine SNP50 Bead Array

The International Sheep Genomics Consortium (ISGC) collected and genotyped samples from 2,819 sheep from 74 breeds as part of a large study into genetic diversity and the impact of selection after domestication ^13^. A DNA sample from the Katahdin ram was provided to the ISGC by USMARC as part of that study and genotyped with the Illumina (San Diego, California, USA) Ovine SNP50 Bead Array. Genotypes for the Katahdin ram were extracted with PLINK ^14^ for subsequent analysis.

### DNA preparation

DNA from the reference animal was extracted by a typical phenol-chloroform-method from 3 ml of thawed whole blood previously stored at -20C ^15^. The concentration and quality of the DNA was initially estimated spectrophotometrically by dissolving in a solution of 10 mM TrisCl, 1 mM EDTA (pH 8.0) and measuring the absorbance at 260 and 280 nm (NanoDrop, Wilmington, DE). Sample degradation and quality was also measured by electrophoresis on 1.5% agarose gel. Approximately 15 to 20 µg of DNA was sent to the sequencing facility (BGI Americas Corporation, Cambridge, Massachusets, USA). The sequencing facility subsequently determined the final sample concentration and integrity by fluorimetry (Qubit, Life Technologies, Grand Island, New York USA) and 1% agarose gel electrophoresis.

### Library construction

Approximately 5 µg of sheep genomic DNA was fragmented by focused-ultrasonication to generate fragments less than 800 bp long (Covaris, Inc. Woburn, Massachusetts USA). The these fragments were used to make a paired-end library according to the manufacturer's instructions (TruSeq DNA Sample Preparation Kit, Illumina, Inc., San Diego, California USA). Paired-end library sequencing was performed on a HiSeq2000 machine (Illumina) with one lane of a flow cell to obtain 100 bp reads from each end of the library insert. After sequencing by synthesis, the raw reads were filtered to remove adaptor sequences, contaminating dimer sequences and low quality reads. The reads have been deposited in the NCBI Sequence Read Archive with accession SRR1013441.

### Alignment

The fastq files from the paired end sequence run for the Katahdin ram were downloaded from the sequencing facility’s ftp site. Once downloaded, the cattle and sheep genomes were indexed for use by the Burrows-Wheeler Aligner (BWA) ^16^, and the BLAST Like Alignment Tool (BLAT) ^17^. The reference assemblies for both UMD3.1 and Oar3.1 were downloaded from the NCBI genomes download site. Repeat information was acquired from UCSC via their genome browser’s download site using:

wget ftp://hgdownload.cse.ucsc.edu/goldenPath/bosTau6/database/nestedRepeats.txt.gz.

The fastq files corresponding to R1 and R2 runs for the paired end libraries were aligned individually using BWA aln, vs UMD3.1 then merged and collated with BWA sampe. The mapping process was repeated for the Oar3.1 reference genome. The resulting sequence alignment map (SAM) files were converted to binary alignment map (BAM) files, and subsequently sorted via SAMtools ^9^. PCR duplicates were marked in the BAM files using the Genome Analysis Toolkit (GATK) ( http://www.broadinstitute.org/gatk/) ^18^. Regions in the mapped dataset that would benefit from realignment due to small in/dels were identified using the GATK module RealignerTargetCreator, and realigned using the module IndelRealigner. The BAM file produced at each of these steps was indexed using SAMtools. The resulting indexed BAM files are made available via the Intrepid Bioinformatics genome browser described in greater detail below.

### Variant detection and filtering

The above mapping efforts produced BAM files for the alignments to both UMD3.1, and Oar3.1, and each BAM file was analyzed for variation against their respective genomes. The GATK UnifiedGenotyper was used with the genotype mode (-gt_mode) flag set to DISCOVERY, and the likelihood model (-glm) flag was set to BOTH in order to identify both single nucleotide variants, and small insertions and deletions. The maximum number of alternate alleles (--max_alternate_alleles) flag was set to allow only 3. Other than those mentioned, default parameters were used. A BED annotation file was created from the nestedRepeats file for the UMD3.1 assembly. The variant call format (VCF) file produced from the dataset mapped to UMD3.1 was filtered to remove any variants that were detected in repeat regions of the UMD3.1 reference assembly using vcftools ( http://vcftools.sourceforge.net) ^19^. Since this was a ram, variants identified on chrX were filtered out of the resulting dataset. This filtered file was used in all subsequent analyses.

### Corroboration of heterozygous genotype calls

Once variants for this animal were identified vs. the cattle reference sequences, a process was created to determine how many of those variants could be corroborated in the alignment to the sheep genome. This required a translation table that listed the corresponding position in the sheep reference for the variants identified vs. the cattle. The process we created was based on the BLAST-like alignment tool, BLAT ^17^. For each of the 3,672,099 heterozygous variants identified in non-repeat regions of the UMD3.1 alignment, 100 bases of reference sequence flanking each side of the variant position was extracted, and a fasta record was created for each variant. These fasta records were collected in groups of 10,000, and BLATed against the sheep genome. The results were output in the BLAST file format. The BLAT result for each fasta record was analyzed, and high scoring pairs (hsps) were selected that contained the variant position and at least 50% of the 201 bases in the fasta record with greater than or equal to 90% identity across the hsp. From within that hsp, it was possible to identify the chromosome and position in the ovine reference that corresponded to the variant being searched.

Since the animal being studied is a ram, any record amongst these heterozygotes that had a corresponding mapping to chrX on Oar3.1 was removed. With this process, we were able to identify corresponding coordinates in the ovine genome for 1,524,297 of the heterozygotes measured in autosomal, non-repeat regions of the bovine genome. Only variants that had exactly one corresponding hsp spanning the fasta record were considered further.

A VCF file was created with the ovine coordinates derived above, the ovine reference allele, and non- ovine reference base identified at each position and was subsequently passed to the UnifiedGenotyper as the –alleles value, and used in genotyping mode (arguments –glm BOTH, -gt_mode GENOTYPE_GIVEN_ALLELES and -out_mode EMIT_ALL_SITES). The results are summarized in Table 1.

Finally, to validate on an independent platform, these Oar3 coordinates were compared with the Oar3 coordinates of the SNPs on the Illumina Ovine SNP 50 chip, and we were able to identify 5283 assays on that chip that genotype positions in the sheep genome corresponding to those we identified as heterozygous.

## Results

Sequencing results for one lane of paired-end reads for the Katahdin ram consisted of 35,917,868 filtered (i.e. clean) reads comprising 35,891,768,800 bases. The average read length and insert size was 100 and 500 bp, respectively with 95.4% of the reads meeting the Q20 quality score. These reads were mapped to the sheep and cattle reference genomes Oar3.1 and UMD3.1, respectively. In total, 56% of reads covered 76% of UMD3.1 to an average depth of 6.8 reads per site ( Table 2). More than 83 million variants were identified by the interspecies mapping, of which 78 million were homozygous and likely represent interspecies nucleotide differences ( Table 3). An aim of this work was to determine how many heterozygous sites in this animal could be identified via interspecies mapping.

**Table 2.** Genome coverage for datasets mapped to reference assemblies.

Measure	Reference genome	
	Oar 3.1	UMD3.1
Bases covered by at least one read	2,502,381,648	2,047,579,163
Fold coverage of covered bases	11.89	6.86

**Table 3.** Genome-wide variants identified in reference assemblies.

Measure	Reference genome	
	UMD3.1	Oar 3.1
Total variants ^a^	83,144,283	16,287,956 ^b^
Homozygous variants	78,137,488	7,122,032
Heterozygous variants	4,837,702	9,128,452
Heterozygous nonRef variants	169,031	37,472
Total heterozygous sites not in repeat regions	3,672,099	N/D ^c^
Heterozygous sites not in repeat regions	3,542,880	N/D
NonRef Hets not in repeat regions ^d^	129,219	N/D

^a^All variants measured on chromosome X were removed from these totals.

^b^The variants identified vs. Oar3.1 that occurred in repeat regions were not filtered out.

^c^Not determined.

^d^NonRef Hets are heterozygous variants where neither detected allele corresponds to the bovine reference allele at that position.

Excluding genome repeat regions and sex chromosomes, nearly 3.7 million heterozygous sites were identified in this animal vs. bovine UMD3.1, representing polymorphisms occurring in sheep. The homozygous variants are also directly informative for comparative genomics studies and may be used to create a catalogue of interspecies variation.

The reads for this sheep were also mapped to the ovine reference genome Oar3.1. The statistics for mapping, and variant discovery are shown in Table 2 and Table 3 respectively. The objectives of this exercise were twofold. 1) to get a rough estimate of the number of interspecies variants that could be measured, and 2) to determine how many of the intraspecies variants could be measured using the heterozygous variants measured against UMD3.1. The total number of variants measured vs. Oar3.1 were in excess of 16.2 million, 9.1 million of those were heterozygotes, all of which would be considered intraspecies. The total number of heterozygous variants detected vs. the UMD3.1 reference was 4.8 million. There are certainly variants identified in both sets that are artifacts, as well as some that would have been missed due to low read coverage (even though they are the same set of reads, in conserved regions, the depth of coverage was lower in the UMD3.1 mapped dataset ( Table 2)). These numbers provide a rough estimate that suggests roughly 52.7% (4.8M/9.1) of the heterozygous variation can be measured via this interspecies approach.

Due to the myriad mapping artifacts that will occur in an interspecies mapping, measurement of putative intraspecies variation with this approach is likely to be the most error prone. To estimate an error rate, an attempt was made to corroborate these heterozygous measurements vs. UMD3.1 using the Oar3.1 mapping result. Using the process described in the Methods, a table was created that listed the positions identified as heterozygotes vs. non-repeat, regions for UMD3.1, and the coordinate of the corresponding position in the Oar3.1 reference. The UnifiedGenotyper was used to genotype the dataset mapped to Oar3.1 at these corresponding coordinates, and the results are summarized in Table 1. Of the called heterozygous variants vs. UMD3.1, it was possible via our method to identify the corresponding position in Oar3.1 for 1,524,297 variants. Of these variants, heterozygotes could be corroborated for slightly more than 80% of them. For the nearly 20% of the variants that could not be corroborated, there are several explanations including, incorrect mapping of interspecies reads, as well as overzealous calls on the part of the UnifiedGenotyper vs. the UMD3.1 mapping, and errors in our process for identification of corresponding positions between the two assemblies. This error rate could be mitigated significantly with either more coverage, or better yet, more animals from the same species. By adding more coverage there will be a benefit to the genotype likelihood models that identify variants. By adding more animals, many of the artifacts that are the result of inappropriate mappings of reads from orthologous regions will manifest themselves as fixed heterozygotes. In fact, if one were to use this approach to perform an association study, many of the genotyping errors will be weeded out by producing high P-value associations. Regardless, this result suggests that of the 3.67 million heterozygous variants identified in the autosomal, non-repeat regions of the UMD3.1 reference, as many, or more than, 2.94 million (3.67M×0.8) of them are legitimate intraspecies variation.

To evaluate the genotype accuracy of the mapping process, we compared genotypes for the Katahdin ram to those from an Ovine SNP50 BeadArray dataset ^13^. The dataset was distributed by ISGC as a PLINK formatted PED file with corresponding MAP file. The PED file was compared to the 1.52 M filtered heterozygous variants to identify 5283 SNPs present in both data sets. It was necessary to convert the genotypes from the UnifiedGenotyper to the Illumina top strand format. Specifically, C/T, and G/T genotypes identified by the UnifiedGenotyper were converted to A/G, and A/C genotypes, respectively. The comparision showed that, 415 of the 5283 BeadArray SNPs had no genotype call for this animal. However, of the remaining 4868 SNPs, 4821 were concordant (99.03%).

This approach to variant detection using moderate coverage whole genome shotgun (WGS) sequence data in species without reference genomes shows a great deal of promise. However when studying only one animal, an estimated 20% error rate for heterozygous variant detection is sufficiently high to suggest caution in using this information in a high throughput analysis pipelines. When implementing analysis pipelines that use variation data, it is common practice to work directly with a distilled list of variants such as in a VCF file, or other list that would provide the variant as well as summary information derived from the mapped dataset. This summary information includes probability likelihoods, and information about the number of times each allele was measured. This information is very useful, but visual inspection of the alignments are a far more effective approach when working to gain an understanding of the quality of the mappings used to derive the genotype. For this reason, the BAM files used as the foundation of this analysis as well as the VCF files derived from them are provided for direct visual interrogation, and use in analytical pipelines.

**Figure 1.**
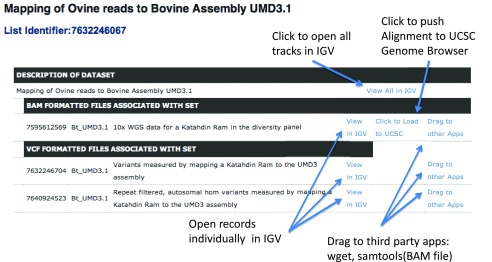
Screen shot of web page with links for the mapped Katahdin Ram reads to the bovine reference genome UMD3.1 (See the Methods for detail description of use).

The BAM and VCF files for the UMD3.1, and Oar3.1 mappings are made available for visualization directly within the IGV modified to use the Intrepid Bioinformatics application programming interface. The links to access the data are provided in Table 4. An example of one of the pages is shown in Figure 1, and the use of the information on the page is described in Supplementary materials. The “View All in IGV” link will display all data mapped to the respective assemblies in IGV.

**Table 4.** Links to pages within the Intrepid Bioinformatics data management system.

**UMD3.1**
http://dx.doi.org/10.13013/J6154F0P
**Oar3.1**
http://dx.doi.org/10.13013/J6WD3XH0

From these web links it is possible to readily view, retrieve, and transfer subsets of these voluminous datasets between autonomous applications such as SAMtools, wget, and other third party applications in a straightforward fashion without requiring researchers to download, or reprocess them.

## Discussion and conclusion

In this work it has been demonstrated that WGS sequence data from one ruminant species (sheep) could be mapped to a mature reference genome from another ruminants species (cattle) diverged 15 to 30 million years ago for the purpose of identifying both inter-, and intraspecies variation in highly conserved genomic regions. Although there is a high quality, annotated reference genome for the sheep, we chose this species for two reasons. First, it provided the opportunity to determine what percentage of intraspecies variation could be identified, and second, it allowed an estimation of how many of the heterozygous variants identified by cross-species mapping could be corroborated against its own genome.

The catalogue of interspecies variation derived from the homozygous variation measured vs. the bovine genome provides insight into the relationship of genome structure and function across different biological species. Although the sequence of one animal is not intended to represent the comprehensive spectrum of alleles within a species, it provides at least one example of alleles that have evolved. Using only one animal as a representative of a species, it will be impossible to determine whether a homozygous variant is between or within species. Irrespective of this limitation whether the variation is inter- or intraspecies, a researcher is given insight as to how much variation is tolerated in an otherwise conserved region. However, for the purpose of intraspecies variation detection, homozygous variants derived from a single animal should be ignored. This problem could be mitigated if more animals were used since that would dramatically increase the number of alleles represented and greatly increase the likelihood that a heterozygote would be measured. The majority of heterozygotes will represent a pair of alleles present in this animal, and therefore intra-species variants. Caution should be exercised, as some of the heterozygotes will be due to artifacts either specific to this approach, or otherwise, of the alignment process. Artifacts specific to this approach include the difficulties inherent in performing variant discovery via interspecies mapping since there is significantly more legitimate variation between the reads and the reference sequence due to interspecies variation. This variation, even within conserved regions, results in significantly lower coverage for interspecies mappings (refer again to Table 2). As for artifacts of the alignment process, if there are two very similar regions of the ovine genome (paralogs, which are orthologous to a unique region in the bovine genome), then reads corresponding to both ovine regions may map to a single region in the bovine genome, and differences between the two ovine regions would appear as heterozygotes in the mapping vs. cattle. It is possible to identify these regions by visual inspection as there would be many heterozygotes within the length scale of a read, and very few homozygous variants in the same region, but it would be difficult to reliably identify these algorithmically. If a population of animals were being analyzed these variants would distinguish themselves as being heterozygous, fixed for all animals in the population.

The approach described here suggests that researchers can pursue important comparative genomics work as well as association studies in species that may be a decade or more from reference genomes. Eventually, our current approach to whole genome analysis, high throughput sequencing followed by mapping to a reference genome will likely be supplanted by technologies that produce, as closely as possible, fully assembled whole genomes for each individual being studied. However, this new reality is still years away.

Finally, a new method for readily viewing, using, and accessing mapped, high throughput datasets and variant files is described. The links allow for access to subsets of data that may be useful to researchers without requiring them to download datasets that may be 10s to 100s of gigabytes in size. Also, the drag and drop formalism presented here introduces a mechanism for user-driven, surface-to-surface interoperation between autonomous applications. This ultimately will allow non-scientific programmers to easily move data between graphical user interfaces of the informatics platforms necessary to do their work.

## Supplementary materials

### Access to BAM and VCF file data via the Intrepid Browser

A representative screenshot of the Intrepid Web page ( http://dx.doi.org/10.13013/J6H41PBX) is shown in Figure 1. By clicking on any of the “View in IGV” links, a java webstart file (.jnlp) is downloaded to the users download directory. That file may be opened to webstart Intrepid’s version of IGV that has been modified to use Intrepid’s application programming interface. The modified source code is posted on our SourceForge site ( http://sourceforge.net/projects/intrepidbio/files/?source=navbar). The source code is also permanently available at 10.5281/zenodo.7523. Once opened, a window will appear from which a user may click to “Load” the data described in the text boxes that correspond to the dataset represented by the link. This will load the dataset and the user may browse to specific locations in IGV. Otherwise, the application will function in a manner consistent with the native application. The “View All in IGV” link may be clicked, and as above will download the .jnlp file which when opened will allow the user to simultaneously load all tracks presented on the web page. The “Click to load to UCSC” link will push a URL corresponding to the BAM file up to the UCSC Genome Browser ^8^, and will add it as a new track onto the appropriate genome. Once loaded, the UCSC browser may be used as it would otherwise. The “Drag to other Apps” link, on Mac OS X machines may be dragged into a terminal window or other third party applications capable of accepting them. The links for the BAM files may be used directly in SAMtools to download subsets of data for use locally, such as by typing

**samtools view –h**

At this point, drag the link to the terminal window, and append the text

**chr13:47,398,414–47,420,508**

producing a command line that resembles

**samtools view –h <dragged link> chr13:47,398,414-47,420,508**

This command, tested using SAMtools-0.1.19, will download in SAM format the SAM header and alignment information for the reads mapping between the coordinates specified on chromosome 13. On a Windows machine, the link may be copied via right click, and pasted into a command window in the appropriate place. These links may be dragged or copied directly into standard versions of IGV available from the Broad Institute or elsewhere by selecting the “File” tab in IGV, and the “Load from URL” option within. Finally, these links may be used with the wget application to download the datasets entirely. It is suggested that wget be used with the –c option in case the download is disrupted for any reason. If the subsets of the files are downloaded via SAMtools, the resulting files will need to be converted (if downloaded in the SAM format) to BAM files and indexed via SAMtools. Downloaded VCF files may be indexed with IGVTools within IGV.
