# Supplementary material for: Mapping whole genome shotgun sequence and variant calling in mammalian species without their reference genomes
Source: F1000Res. 2014 Feb 10;2:244. Originally published 2013 Nov 14. [Version 2] doi: 10.12688/f1000research.2-244.v2 (PMC4103496; doi:10.12688/f1000research.2-244.v2)
Supplement: Peer review history

# Peer review: Referee response for version 2

**DOI:** 10.5256/f1000research.3783.r3571

**Published:** 2014-04-03

**Authors:** Claire Wade

**Affiliations:** 1 Faculty of Veterinary Science, University of Sydney, Sydney, Australia

The authors have adequately addressed my reservations on the article.

I have read this submission. I believe that I have an appropriate level of expertise to confirm that it is of an acceptable scientific standard.

# Peer review: Referee response for version 2

**DOI:** 10.5256/f1000research.3783.r3870

**Published:** 2014-04-02

**Authors:** María Ávila-Arcos

**Affiliations:** 1 Center for GeoGenetics, National History Museum of Denmark, Copenhagen, Denmark

The paper reports in a very thorough and clear fashion the bioinformatic steps taken to map short reads to a reference genome of another species and call potential variants. The way the data is made available and the tools provided for such are great features in this report. 

Although the bioinformatics are solid and clearly described, the actual applicability and perspective for this type of approach is limited, as the authors themselves acknowledge. 

If anything remains to be tested it is if different parameters in the mapping algorithm, in this case BWA (e.g. -l and -i parameters), or even using another more suitable mapper (for example GEM) could either help to retrieve more variants, or reduce the FDR (Ideally a combination of both!)

Overall, I agree this paper reports an interesting exercise, however other researchers facing a similar problem (lack of same species’ reference genome) need to take into consideration species-specific genomic features of source and reference genomes and interpret results with caution.

I have read this submission. I believe that I have an appropriate level of expertise to confirm that it is of an acceptable scientific standard.

# Peer review: Referee response for version 1

**DOI:** 10.5256/f1000research.2842.r2733

**Published:** 2014-01-16

**Authors:** Claire Wade

**Affiliations:** 1 Faculty of Veterinary Science, University of Sydney, Sydney, Australia

The majority of the paper contains well conducted bioinformatic research. I would ask for a small amount of laboratory work and the editing of one section.

To infer a rate of validation of the SNP discovery, it would be useful to see a small subset of identified SNP (10-20 should be sufficient) randomly chosen from the discovery set genotyped using a different method (e.g. Sequenom) in the discovery animal DNA, and ideally a small cohort of animals of the same breed.

The section "Access to BAM and VCF file data via the Intrepid Browser" is superfluous to the major paper and could be removed and replaced with a reference link to the paper and a second link describing access instructions.

I have read this submission. I believe that I have an appropriate level of expertise to confirm that it is of an acceptable scientific standard, however I have significant reservations, as outlined above.

# Response

**Published:** 2014-02-10

**Authors:** Ted Kalbfleisch

**Affiliations:** University of Louisville Health Sciences Center, USA

Thank you for your comments and suggestions.  Please find below a description of our efforts to address them.  We hope that your find them satisfactory.

- *“[I]t would be useful to see a small subset of identified SNP (10-20 should be sufficient) randomly chosen from the discovery set genotyped using a different method (e.g. Sequenom) in the discovery animal DNA, and ideally a small cohort of animals of the same breed.”  *
  We were able to acquire an Illumina Ovine 50k SNP Chip dataset for the animal in this study.  We identified 5283 assays on the chip that corresponded to positions predicted in our study as polymorphic in sheep.  The process for selecting variants to include on a SNP chip is rigorous.  The fact that assays for these variants were included on the chip indicates that they are demonstrated to be either informative with respect to disease risk, or have high allele frequencies in sheep.  When compared, and excluding those assays that produced no genotype call, 99.03% of the genotype calls were in agreement between the Ilumina platform, and our approach. 
- As for the section in the paper  “Access to BAM and VCF file data via the Intrepid Browser”, we agree that this section does not directly support the paper’s findings.  This section has been moved from “Methods” to “Supplemental materials”.
